# Local Cartilage Trauma as a Pathogenic Factor in Autoimmunity (One Hypothesis Based on Patients with Relapsing Polychondritis Triggered by Cartilage Trauma)

**DOI:** 10.1155/2012/453698

**Published:** 2011-10-27

**Authors:** Carlos A. Cañas, Fabio Bonilla Abadía

**Affiliations:** Rheumatology Unit, Fundación Valle del Lili, ICESI University, Avenida Simón Bolívar Carrera 98 No.18-49, Cali, Colombia

## Abstract

In the recent years, it has been of great interest to study the binding mechanism between the innate and adaptive immune responses as interrelated processes for the development of multiple autoimmune diseases. Infection has been a well-known trigger of autoimmunity and trauma has been related as well too. Cryptogenic antigens release, recognition of pathogenic structure, and metabolic changes generated by both stimuli begin an inflammatory process which in turn activates the immune system amplifying T and B cell responses. The development of relapsing polychondritis after trauma may have a direct association with these events and in turn probably trigger autoimmune phenomena.

## 1. Introduction

Proteins that are hidden in the tissues and by different events such as trauma begin to be recognized and attacked by immune system are called cryptogenic antigens, so it starts a process initially with innate and later with acquired immunity mechanisms. An example of this condition is the sympathetic ophthalmia (SO) where breaching of systemic ocular barriers compromises the relative immune privilege of the eye and causes sensitization to previously sequestered uveoretinal antigens [1]. A similar mechanism is observed in relapsing polychondritis (RP) started by local cartilage trauma triggering an immune response against cartilage in distant sites of the body and even in noncartilaginous tissues [2].

## 2. Lessons Learned of SO

This disease is precipitated by ocular trauma to one eye, followed by destructive inflammation in the nontraumatized or “sympathizing” eye [3]. It is thought that antigens released from the traumatized eye find their way into the draining lymph node and generate systemic immune response [4]. An accompanying infection may provide an adjuvant effect, although severe endophthalmitis, which quickly destroys the injured eye and eliminates the source of antigen, may actually lessen chances of developing the disease [5]. In uveitic disease, it is believed that T cells are capable of recognizing retinal antigens by microbial stimuli that may be immunologically similar in structure to their cognate retinal antigen (antigenic mimicry). Microbial components also interact with innate pattern recognitionreceptors (PRRs) on antigen-presenting cells, generating “danger” signals that are necessary to elicit inflammatory reactions. Following exposure to an uveitogenic stimulus, circulating retinal antigen-specific cells become activated and acquire effectors function [5]. In the above examples, it is postulated that the retinal antigen recognition distance may be submitted by local effects, but it is obvious to assume that there is not a similar type of tissue throughout the body. Therefore, we cannot rule out that it can presented by a systemic effect. 

## 3. Local Cartilage Trauma as a Triggering Factor of RP

RP is a rare immune-mediated disease of unknown etiology which is associated with inflammation in cartilaginous, especially hyaline cartilage throughout the body.

Possible mechanisms involved are an autoimmune background and a trigger factor such as cancer, infection as hepatitis C virus [6], or drugs (e.g., anti-TNF) [7]. In some cases, recently reported cartilage trauma may be a trigger of the disease in a susceptible person [8–10]. Puncture or other type of trauma with or without presence of foreign material in the cartilage and often associated with infection could trigger an autoimmune disorder by exposing unusual cartilage matrix protein antigens [11].

Also in this case like SO, probably the proteins that are hidden in the tissues would be exposed by factors such as trauma and begin to be recognized by the immune system as foreign, triggering processes for their elimination initially with innate and later with acquired immune mechanisms. This process has not been clearly defined; however, several reported cases of RP have shown an association between mechanical insult to cartilage and the development of more local cartilage inflammation and even the involvement of distant cartilaginous tissues [2].

One hypothesis comes out from these conditions and interrelate innate immune response, and the activation of the adaptive system may be mediated by PRR of the innate immune cells such as Toll-like receptors (TLRs) and NOD-(nucleotide-binding oligomerization domain-)like receptors (NLRs). TLR family consists of 10 receptors that are divided into membrane and endosomal localization and which allow for recognition and response to pathogen-associated molecular patterns (PAMPs) representing diverse microbial products (lipopolysaccharide, flagellin) of most pathogens [12, 13]. This could explain the significant role of infection as a coadjuvant factor in OS and in our cases of RP. Infection is a well-known contributing factor for the development of autoimmunity [14, 15]. NLR family members are cytosolic sensors of microbial components and danger signals such as damage-associated molecular patterns (DAMPs) which represent common metabolic consequences of infection and inflammation released during the tissue damage and cell lysis and include high mobility group box 1 protein, alarmins, heat shock proteins, and uric acid [16, 17].

These two ways of pattern recognition contribute to making the link between innate and adaptive immune response through the production and expression of IL-1B and IL-18 generating an inflammatory response and acting on lymphocytes in several pathways including upregulating IL-2 receptor expression, which in turn prolongs survival of T cells and increases B cell proliferation and production of antibodies [18, 19]. IL-1B and IL-18 also play a crucial role in driving the differentiation and amplification of Th17 and Th1 cells, respectively [20, 21]. Thus, in general, these interleukins amplify T and B cell responses and might serve as a crucial link translating into adaptive immune responses [18]. In the RP triggered by local trauma, the inflammatory reaction may lead to the production of autoantibodies and autoreactive T lymphocytes against cartilage structures (e.g., collagen type II) which in turn could develop chondritis at other sites [22].

## 4. RP and Systemic Autoimmune Phenomena

It is well known and reported in the literature that about 30% of the patients with RP have additional autoimmune or hematological diseases, most frequently systemic vasculitis, rheumatoid arthritis, myelodysplastic syndromes, or systemic lupus erythematous [23].

Recently, we reported a comparative analysis of 18 patients diagnosed with RP with or without tissue trauma (7 versus 11 patients, resp.) and evaluated the development of the systemic autoimmune phenomena. The results showed that the patients with previous local trauma lead to a greater systemic autoimmune response shown by the development of antinuclear antibodies, rheumatoid factor, antineutrophil cytoplasmic antibodies, autoimmune thyroiditis, rheumatoid arthritis, and vasculitis [2]. Also included in this last group, the description of an RP case induced by local cartilage trauma in the ankle with later development of central nervous system compromise manifested as a hypertrophic pachymeningitis, PR3 ANCAs-positive vaculitis outside of the complete clinical expression of a Wegener's granulomatosis with request for aggressive treatment with cyclophosphamide and rituximab given severity of the condition and poor initial response to treatment [24]. 

**Figure 1:**
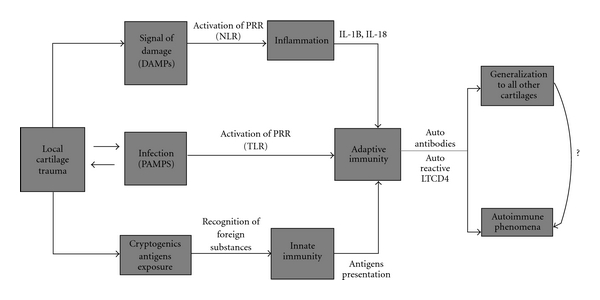
Local cartilage trauma initiates an inflammatory process with or without infection as coadjuvant as well as activation of the innate and adaptive immunity through induction of PAMPs, DAMPs, and cryptogenic antigens which are recognized by PRR (TLR, NLR) that results in the production of IL-1B and IL-18. These interleukins amplify T and B cell responses and might serve as a crucial link into adaptive immune responses. PRR, pattern recognition receptors; TLR, Toll-like receptors; NLR, NOD-like receptors; LTCD4, lymphocytes T CD4.

Figure 1 outlines the hypothetic steps in the pathogenesis of autoimmunity in patients with local cartilage trauma and development of RP.

## 5. Conclusions

Local cartilage trauma with or without infection triggers an immune processes directed against cryptogenic antigens that conduce to inflammation of cartilage both local and later in other sites of the body. By diverse mechanisms which may be implicated by PRR such as TLR and NLR, a systemic autoimmune phenomena is induced. The presentation of RP after local cartilage trauma may influence the presence of this systemic autoimmunity.
